# Psychosocial and bioethical challenges and developments for the future of vascularized composite allotransplantation: A scoping review and viewpoint of recent developments and clinical experiences in the field of vascularized composite allotransplantation

**DOI:** 10.3389/fpsyg.2022.1045144

**Published:** 2022-12-15

**Authors:** Martin Kumnig, Sheila G. Jowsey-Gregoire, Elisa J. Gordon, Gabriele Werner-Felmayer

**Affiliations:** ^1^Department of Psychiatry, Psychotherapy, Psychosomatics and Medical Psychology, Center for Advanced Psychology Transplantation Medicine (CAPTM), Medical University of Innsbruck, Innsbruck, Austria; ^2^Department of Psychiatry and Psychology, Mayo Clinic Rochester, Mayo Graduate School of Medicine, Rochester, MN, United States; ^3^Department of Surgery, Vanderbilt University Medical Center, Nashville, TN, United States; ^4^Institute of Biological Chemistry and Bioethics Network Ethucation, Medical University of Innsbruck, Innsbruck, Austria

**Keywords:** vascularized composite allotransplantation, psychosocial, bioethics, quality-of-life, peer education, patient reported outcomes

## Abstract

Vascularized Composite Allotransplantation (VCA) has evolved in recent years, encompassing hand, face, uterus, penile, and lower extremity transplantation. Accordingly, without centralized oversight by United States Organ Procurement and Transplantation Network (OPTN) or European Programs, centers have developed their own practices and procedures that likely vary, and accordingly, present different levels of rigor to the evaluation process, internationally. The importance of psychosocial factors in the selection process and treatment course has been widely recognized, and therefore, several approaches have been developed to standardize and guide care of VCA candidates and recipients. We propose to develop an international multidisciplinary platform for the exchange of expertise that includes clinical, patient, and research perspectives. Patient perspectives would derive from peer education and the assessment of patient-reported outcomes. To establish a foundation for such a platform, future research should review and combine current VCA protocols, to develop the ethical framework for a standardized psychosocial evaluation and follow-up of VCA candidates and recipients. This review presents a comprehensive overview of recent results in the field of VCA, developments in structural aspects of VCA, and provides viewpoints driven from clinical experience.

## 1. Introduction

### 1.1. Current standards and framework development

Vascularized composite allotransplantations (VCA) have moved from a purely experimental option for a small number of patients, to comprising the standard of care of VCA recipients at some institutions internationally (Kumnig and Jowsey-Gregoire, 2016; Hautz et al., 2020). However, this transition has occurred without a detailed, comprehensive, and objective investigation of psychosocial and bioethical factors (Kumnig et al., 2012, 2014a,b; Kumnig and Jowsey-Gregoire, 2016). Ensuring that psychosocial and bioethical implications of VCA transplants are established within the VCA field is highly important; however, standardized protocols for the evaluation and follow-up VCA patients are still evolving (Kumnig et al., 2022). VCA procedures are primarily life-saving, such that quality of life (QOL) comprises central outcomes. The most important development for the VCA field is an emerging recognition that the pre-and post-transplant psychosocial evaluation and treatment is an integral part of any VCA transplant program, and that the identification of at-risk patients and those requiring ongoing counseling is a primary focus of assessment and treatment procedures (Kumnig et al., 2012, 2014a,b; Kumnig and Jowsey-Gregoire, 2016). The psychosocial risk assessment encompasses whether the patient understands the potential surgical complications, the risks of immunosuppression, the potential for rejection and graft loss (Goldade et al., 2011), and the need for adherence with the therapeutic regimen (Matas et al., 2002). Appropriate patient selection is important because of patients’ risk of developing a psychiatric disorder or because patients may be struggling with psychosocial burdens before transplantation as well as during the post-transplant period (Rosenberger et al., 2012; Pither et al., 2014).

Psychosocial factors are important elements in the assessment and follow-up care for VCA and require multidisciplinary evaluation protocols. The Chauvet workgroup has been convened with membership from a number of transplant centers to address these issues and to call for ongoing global research collaboration. A multicenter research network, a consortium of collaborating VCA centers, would share similar evaluation approaches so that meaningful research on psychosocial variables could inform the transplant community and patients about psychosocial factors relevant for optimal VCA outcomes (Kumnig et al., 2022).

Because of the extant global diversity in psychosocial evaluation and follow-up routines in VCA, current and future research will need to guide the field regarding this question: What are current standards and what important psychosocial aspects must be considered when implementing or re-structuring a VCA program at a transplant center? Moving one step forward, directly into the psychosocial evaluation and follow-up process, this perspective will particularly address the importance of the assessment and elaboration of ‘exit strategies’ with candidates planning to undergo VCA or patients who already have been transplanted and their strategies to cope with potential graft loss during the transplant course. This topic is especially important for this field and there is no sufficient explanation as to why this has been neglected for such a long period of time. This important topic is under examination in the kidney-pancreas community of practice *via* American Society of Transplantation (AST), so that future developments in the field of VCA could orientate to such protocols (Alhamad et al., 2022).

### 1.2. Peer education and consultation concept in vascularized composite allotransplantation

Considering the small number of global VCA centers (about 50 centers worldwide) and VCA recipients (under 200 recipients of different VCA procedures to date, worldwide; Kinsley et al., 2020), international collaborations have sought to bring health professionals and patients together in other clinical contexts (Magill et al., 2019). Therefore, future research will need to address the possibilities of peer education in the field of VCA, educational programs that bring healthcare professionals together as well as connecting patients worldwide (particularly because of the small number of cases). Although international collaboration has been an essential part in the history of VCA, it is still difficult to share experiences and to bring different perspectives together. Thus, the concept of an international platform for experts (e.g., Chauvet Research Group; Kumnig et al., 2022) and patients could lay the foundation to provide this essential global connectivity.

Further, low-threshold consultation concepts for post-transplant VCA patients, for example by routinely assessing patient-reported outcomes (PROs) could guide a targeted expert counseling. That will help to address the specific needs of these patients: e.g., information-related questions, psychosocial issues (e.g., depressive developments, adherence problems). Moreover, such assessment routines could help to identify patients at risk, offering a specific consultation first, evaluate by routinely collecting psychosocial outcomes (PROs), and decide whether additional intensive care may be needed.

## 2. Important psychosocial issues identified by recent research initiatives

### 2.1. Psychosocial stability, financial, caregiving, and family responsibility

First, it must be noted that there are financial differences between European countries such as the United Kingdom and France, compared to the United States, which may account for differences in what is considered a contraindication for VCA. Finances are generally not considered a significant barrier to healthcare in European countries due to caregiving, hospital, and post-transplant state-sponsored support, like in United States, where the family is commonly expected to provide some financial support to help the patient meet co-payments for care along the continuum of pre-to post-transplant periods (Wainright et al., 2022). While financial issues do not comprise an absolute contraindication to VCA in Europe, such issues could potentially be construed as an absolute contraindication to VCA procedures in the United States. Exemplary in France, there is no financial payment expected from the patient for the operation, hospitalizations, and post-transplant care for the rest of the life of the patient. In the United States, however, insurance companies do not yet cover VCA procedures as they are still considered ‘experimental’ until more data are collected about patient outcomes. Accordingly, patients may find it challenging to access and cover the financial costs of VCA treatment, immunosuppression, and support themselves in the post-transplant period. Thus, establishing insurance coverage for VCA in the United States could help to expand VCA transplant procedures.

### 2.2. Coping history, adherence history, and issues of substance abuse

There is early consensus among global VCA centers that active substance use at the time of evaluation is a contraindication to any VCA procedure (Jowsey-Gregoire et al., 2016). However, the question is raised whether a patient who undergoes recommended treatment for addiction can become a candidate in the future. It remains to be determined whether active substance abuse should be a relative and temporary contraindication to VCA and if in that case recommendations for substance use treatment should be made to improve the patient’s candidacy.

Recent research suggests that a period of longitudinal follow-up should be part of the protocol, to allow re-assessment and follow-up at multiple time points prior to VCA. The optimal proposed follow-up period was for 1 year from the time of assessment for the potential VCA procedure (Kumnig and Jowsey-Gregoire, 2016).

Particularly in the case of patients with a history of non-adherence with medical recommendations, the evaluation and development of coping and (non-)adherence is important. It is proposed that a psychometric instrument may help with tracking adherence with immunosuppressive medications in the post-transplant period (Jowsey-Gregoire et al., 2016). Recent research also highlights that use of modern technologies (phone apps, digital wrist-worn devices) has the potential to empower the patient and should possibly be considered to assist the patient with adherence to medications. The clinical psychological assessment still remains the most important tool to take care of non-adherence (Kumnig and Jowsey-Gregoire, 2016).

### 2.3. Psychosocial contraindications to vascularized composite allotransplantation

Most centers consider risk factors in VCA to be relative, and potentially modifiable (Jowsey-Gregoire and Kumnig, 2016; Jowsey-Gregoire et al., 2016; Kumnig and Jowsey-Gregoire, 2016). Certain psychiatric disorders, such as severe personality disorders, active substance abuse (including nicotine), schizophrenia, chronic depression, and bipolar disorder are considered as risk factors for poor outcomes across many VCA centers (Jowsey-Gregoire et al., 2016). Unreasonable expectations, a history of non-adherence, relational difficulties with the team, and lack of social support are also considered risk factors for a poor outcome (Jowsey-Gregoire et al., 2016). The conclusion of past Chauvet meetings that active psychotic illness is an absolute contraindication to any kind of VCA procedure, can be emphasized here as one central absolute contraindication (Jowsey-Gregoire et al., 2016). Substance abuse was felt to be a relative contraindication and it was offered that chemical dependency treatment for those with active substance abuse can improve a patient’s candidacy for VCA. Chauvet meeting members considered strong social support for this particularly vulnerable population as equally important in assisting with abstinence from substances. The Chauvet meetings noted that patients with substance use issues who have strong social support do better after solid organ transplantation and that this model can be applied to VCA as well (Jowsey-Gregoire et al., 2016).

In terms of appropriate psychological acceptance of the disfigured body (in case of hand and face transplantation), research found that some patients with bodily disfigurement become social recluses, indicating a possible maladaptive coping mechanism (Sarwer et al., 2022). Therefore, many VCA centers raised the question, whether VCA candidates should be required to demonstrate a period of appropriate social interaction before undergoing the VCA procedure. This requirement may necessitate an understanding of the patient’s baseline social behaviors prior to bodily disfigurement (Sarwer and Crerand, 2008; Sarwer and Spitzer, 2012; Sarwer et al., 2022).

Any one risk factor, if severe enough, may constitute an absolute contraindication. The presence of multiple risk factors may also constitute a prohibitive risk. In particular, severe personality disorders, active substance abuse, schizophrenia, and unrealistic expectations would typically be considered risk factors that would be associated with a decision not to approve candidates for VCA transplantation.

### 2.4. Evaluation for vascularized composite allotransplantation and follow-up after transplantation

The psychosocial assessment is considered the principal means of assessing personality, emotional preparedness, cognitive status, coping style, motivation and expectations, and social support. Psychiatrists, psychologists, and social workers are typically involved in this evaluation process.

Research recommends that candidates undergo the psychosocial evaluation after meeting with the surgical and medical team members (Kumnig et al., 2012, 2014b; Jowsey-Gregoire and Kumnig, 2016). This sequence may allow the psychosocial healthcare professional to assess how well the candidate understands the risks and the benefits of the VCA procedure following a thorough discussion with the medical, surgical, and psychosocial teams.

### 2.5. Sex and sexuality, esthetics, and occupation

The first point made in investigations was that sex and sexuality in VCA are not openly discussed by the transplant team (Mills et al., 2020). Therefore, little is known about how patients are expressing their sexuality after a VCA procedure. This is particularly important for women considering uterine transplantation and men considering penile transplantation. In the case of hand and face transplantation, embedded in this notion is the fact that they have lived through the stigma of looking different; they may have suffered social isolation. Therefore, transplant teams should consider the role of social identity and how patients perceive their social identity in relation to their sexuality. The question remains: how does one measure esthetic outcomes in VCA? Transplant teams must rely on the subjective standpoint of the patient but we use objective standards to judge this by.

The value of social support for maintaining and restoring good health is well established, and the assessment of social support has been an aspect in the screening of transplant patients for some time, including VCA patients (Ladin et al., 2019a,b). Nonetheless, the concept of social support in transplantation has been subject of several critiques including the lack of a clear definition, the lack of agreement on a method for assessment, and debate over its use as a criterion for exclusion in patient selection. Future research should investigate the role of social support in upper extremity VCA, and evaluate how differences between SOT and VCA may influence the meaning and value of social support for recipients, and suggest ways in which social support may be better assessed pre-transplant and strengthened post-transplant in VCA.

Research also show that assessing the success of VCA functionality and the ability to return to work are important (again, in case of hand and face transplantation), and that it is necessary to consider the pre-morbid occupational function of the patient in order to gain perspective on occupation as an index of success of the transplantation (Smith and Cendales, 2019). The salient consideration is not necessarily whether the patient has gainful employment but rather how occupation is part and parcel of general social functioning. Also playing a critical role in the function of the family, as well as engaging in non-gainful employment such as volunteer work. An equally important consideration for transplant evaluation is whether the patient’s occupation changed from pre- to post-transplant; and whether patients were required or underwent job retraining to re-enter the workforce (Bramstedt, 2018). We recommend that patients should indicate prior to transplantation how personally important employment is to them, in order to establish a baseline.

### 2.6. Special psychosocial issues in uterine transplantation

Uterine transplantation differs from other forms of transplantation (solid organs or VCA) in many ways: (i) it gives women with absolute uterine factor infertility a chance to realize their wish for a biological child; (ii) the clinical outcome is not only relevant for the patient but also for the child conceived; (iii) transplantation is transitory and its endpoint is marked by graft hysterectomy; (iv) the surgical success rate is defined by a technically successful transplantation with a subsequent regular menstrual pattern potentially allowing for pregnancy and live birth of a child to round off surgical success (Brännström et al., 2021); (v) even after successful uterus transplantation pregnancy may still fail; (vi) about two-thirds of donors are live donors due to disadvantages of deceased donation (Kisu and Banno, 2022); (vii) the surgery is even more invasive and complicated for the live donor than for the recipient and for both patients there are high surgical complication rates (Brännström et al., 2021); (viii) additional risks for live donors include possible familial pressure to donate and reduced quality of life due to hysterectomy and sexual dysfunction (Kisu and Banno, 2022); and (ix) thus far, children born as a result of uterus transplantation were born prematurely at a high rate and with an associated high proportion of respiratory distress syndrome (Brännström et al., 2021).

Medically assisted reproduction is a highly complex field not only clinically but also ethically. Some procedures are highly invasive for women and the children they conceive with the technologies applied. This is certainly even more pronounced in the context of assisted reproduction after uterus transplantation. Patients in this scenario are thus transplant patients as well as patients undergoing fertility treatment to fulfil their wish for a biological child. While this wish can be considered “natural,” it is also highly shaped by sociocultural context rendering those patients particularly vulnerable.

The new recommended framework of preoperative psychological evaluation has been published (Järvholm et al., 2018; Wainright et al., 2018) and was presented at the first three Chauvet meetings. Representatives of uterine VCA centers suggest addressing the following psychosocial domains prior to transplantation (in addition to the general assess psychosocial aspects in VCA, e.g., psychopathology, adherence, social support, coping skills, substance abuse, knowledge of the procedure, motivations, informed consent, etc.): donors’ family planning, coping with childlessness, the couple’s relationship to the donor, and motivation for donation. The last three Chauvet meetings raised key questions about critical psychological events after uterus transplantation, including: who should transplant teams favor as a donor, or when is the appropriate time to stop attempts to achieve pregnancy and remove the uterus, and how can transplant teams create supportive strategies that help patients to deal with graft loss (‘exit strategies’) as well as pregnancy loss?

## 3. Quality-of-life assessment for vascularized composite allotransplantation

Quality of life (QOL) is considered the most important domain for study both before and after transplantation (Feurer et al., 2004; Jensen et al., 2012; Kumnig et al., 2014b). The Chauvet participants recognized QOL as a relative concept, both within and across cultures, and transplant teams must take into account both of those domains of QOL universally held, and those valued for their uniqueness to a particular environment (Verdugo et al., 2005; Petruzzo and Dubernard, 2011; Prieto et al., 2016). At present, there are no instruments uniquely devoted to evaluating these domains in VCA, but there is wide acceptance that depression and anxiety should be assessed at regular intervals. Recent investigations highlight how important it is to examine QOL from the patient perspective, thus PRO assessment has gained increasing traction in the transplant field. The need for different PROs and specific questions/assessment for different types of grafts (hand, face, and uterine) has already been noted by the United States Department of Defense, which issued a call for proposals on this topic earlier in 2022. QOL assessments should also account for body image adjustments because some patients state they want to feel physically whole, and some report a greater need for a good physical match of the graft. Future research should consider the importance of graft functioning to the patient. For example, some patients are satisfied with a limb that is less functional. Also, the patient’s age, gender, race/ethnicity, marital status, education levels, etc., may play an important role in QOL assessment because older persons may differ from younger ones on the importance of different psychosocial domains. In addition, the providers’ perspective likely differs from the patients’ experiences. The development of a VCA-specific QOL protocol/instrument should consider the following domains: importance of impact of graft outcomes on relationships; in the case of hand transplantation: being able to touch, hold love ones; in case of face transplantation: ability to express emotion; sense of connection with the graft; fear of medical complications; and fear of graft rejection (Smith and Cendales, 2019; Bound Alberti et al., 2022).

## 4. Informed consent and potential graft loss (‘exit-strategies’) in the evaluation and follow-up course

As generally known from the field of solid organ transplantation, also in VCA the evaluation and follow-up course are central elements for the success of the procedure. However, in the VCA context, informed consent is of particular importance for several reasons. First, patients need to consider whether the potential benefits outweigh the risks given that the goal of VCA is on restoring functionality and quality of life, rather than on saving life (Cooney et al., 2018). Second, relatively little is known about the psychosocial outcomes of different VCA organ recipients because of the small numbers of patients undergoing VCA. In the United States, for example, VCA transplant programs perform very few VCA transplants per year, making it nearly impossible to collect research data on a sample of recipients large enough to generate meaningful analyzes. Consequently, little is empirically known about patients’ experiences of key elements of the informed consent process (i.e., information disclosure, comprehension, voluntary decision-making) that could help potential recipients make more informed treatment decisions about undergoing VCA and help families decide whether to authorize VCA deceased donation of their loved one’s organs. One study assessed the availability and quality of VCA public education materials (Van Pilsum Rasmussen et al., 2020), and found that educational materials addressed upper extremity and face transplants more commonly than other VCA types, and that few materials identified patient populations who could benefit from VCA and the requirements for authorizing VCA donation. The study concluded that currently available VCA public education materials did not adequately educate the public (Van Pilsum Rasmussen et al., 2020). Similarly, a focus group study found that members of the public had little knowledge of VCA; reported information needs about who could donate, who needs a VCA, and the success rate; and maintained misunderstandings of VCA (Ferzola et al., 2022). A research study conducting interviews and focus groups among individuals with upper extremity amputations and individuals pursuing or had received an upper extremity VCA about decision-making to pursue VCA found that participants desired extensive information about upper extremity VCA in order to make decisions (Gacki-Smith et al., 2022). Many reported that their decisions in favor of pursuing upper extremity VCA were based on the prospect of regaining functionality and its associated independence, increasing social and physical confidence, and enabling more active parental involvement in childrearing; by contrast, those against pursuing upper extremity VCA reported concerns about their health or limb functioning becoming “worse off,” the rigorous rehabilitation process, and having adapted to life without upper limb(s) (Gordon, 2022). Further, individuals maintained various definitions of “success” of upper extremity VCA (Kinsley et al., 2021; Downey et al., 2022). Other research has examined patient’s perceptions of the risks and benefits of upper extremity VCA (Jensen et al., 2014). In sum, these studies highlight the need for VCA transplant programs to inform potential upper extremity and other recipients about VCA as part of the informed consent process. Toward that end, a publicly available neutral educational website, *Within Reach*, has been developed to provide patients, families, and healthcare providers with patient-centered information to make informed decisions about upper extremity VCA (Gordon, 2022). While these aforementioned studies focus prospectively on attaining a VCA organ, little attention has been devoted to “exit” strategies for responding to VCA graft loss. Specifically, the informed consent process should address whether recipients will need to undergo the removal of the VCA face or upper extremity or penis, the potential for re-transplantation compared to amputation or prosthetic care as options, as well as strategies and resources available to assist recipients in coping with graft loss (Smith and Cendales, 2019). Particularly because of the life-saving character of face VCA transplantations, these procedures need to be repeated (re-transplantation after graft failure; Kauke et al., 2021). Particularly because of the life-saving character of face VCA transplantations, these procedures need to be repeated (re-transplantation after graft failure). Until now, two patients have had face re-transplantation, one in France and one in the United States (Lantieri et al., 2020; Kauke et al., 2021). One case was notable for significant pain and loss of facial motion prior to removal of the graft. The patient then experienced visual hallucinations due to sensory deprivation after the graft was removed. Following re-transplantation, the patient-reported anxiety but his symptoms gradually improved and he reported reasonable quality of life and was able to resume work on a part time basis 2 years after transplant (Lantieri et al., 2020). A second facial re-transplantation was reported in 2021. Prior to re-transplantation, the patient was reported to have facial tightness, pain, and contraction with functional limitations in eating, drinking, and speaking and was re-transplanted without a period of time in which the donor graft was removed prior to re-transplantation (Kauke et al., 2021). These cases demonstrate both the feasibility and challenges of re-transplantation for face transplant recipients. The very significant loss of function, sensory deprivation if the graft is explanted prior to re-transplantation, and management of pain are notable points. The extensive nature of the allografts in these cases would suggest that other alternatives would not have been feasible. Significant advances in candidate selection, technology, operative technique, post-transplant care, and immunosuppressive management have contributed to the tremendous expansion of the field. Despite these recent achievements, face VCA transplant require complex surgical techniques, excellent immunosuppressive management, and well-established evaluation (limited allograft donor pool) and follow-up protocols as well as continued collaborative and multidisciplinary research efforts (Lantieri et al., 2020; Diep et al., 2021).

## 5. Bioethical considerations

There are numerous bioethical issues in VCA beyond respecting patients’ autonomy by ensuring comprehensive informed consent as outlined above. Specifically, key ethical challenges include: management of the intense doctor-patient relationship, establishing fair patient selection and transparency of outcomes, maintaining donor registries, collecting and sharing data to advance the field of VCA, disparities, and gaining trust and support for the transition of VCA to becoming standard of care covered by insurance. Another issue is VCA procurement as there is no standardization or allocation system in place and it is usually done *ad hoc*. Policies and methods are needed to protect dignity of deceased donors and next of kin in the procurement process (Magill et al., 2019). Determining the needs of particular patients and whether VCA is the right treatment for them or if available alternatives would be more helpful also remains a challenge.

As pointed out by various authors, public awareness about VCA must be raised in order to support public trust (Caplan et al., 2019; Magill et al., 2019; Van Pilsum Rasmussen et al., 2020). While VCAs are legally considered organs in the United States since 2014, in the Eurotransplant member states (Samuel, 2016).

A major bioethical issue of VCA transplantation is the vulnerability of recipients with regard to functional and visible outcome as well as their hopes and expectations for the benefit of the challenging treatment. Other reasons why VCA recipients are considered to be particularly vulnerable is the temporary celebrity status they may acquire (Caplan et al., 2019) as well as potential harms incurred by providing an economy of fame (Magill et al., 2019). Patient advocacy has been identified as an important countermeasure to deal with vulnerability. It may be possible to support recipients’ participation in their healthcare and helps to provide “ethical protection for both patients/candidates and transplantation teams who share the universal predisposition to self-justification and self-deception” (Benedict, 2022). Patient advocacy also safeguards living donors (Benedict, 2022). For example, the United States Organ Procurement and Transplantation Network (OPTN) requires the involvement of an independent living donor advocate (ILDA) to protect the best interests of a person who is willing to donate an organ while alive (Benedict, 2022). This is of particular interest also for the VCA context, as living donation for certain VCAs is increasingly practiced (Beederman et al., 2022), and is of particular interest for pediatric VCA (Pomahac et al., 2018).

An important bioethical issue pertains to racial/ethnic disparities in VCA recipients. In the United States, VCA recipients appear to be more White patients than Black or Hispanic patients across VCA organ types. For example, among uterus transplant recipients, 89.2% were White patients, 5.4% were Asian patients, 2.7% were Black, and 2.7% were multiracial (OPTN, 2022). However, the racial/ethnic profile of UE VCA recipients is not clear because many recipients are reported as “unknown” (OPTN, 2022), which precludes the analysis of racial/ethnic disparities in VCA receipt. For example, among all bilateral upper limb recipients in the United States there were *n* = 9 White, *n* = 1 Black, and *n* = 9 unknown race/ethnicity recipients; among all unilateral upper limb recipients in the United States, there were *n* = 4 White, *n* = 1 Hispanic, and *n* = 13 unknown recipients. One pediatric bilateral upper limb transplant recipient was Black. Little is known about factors contributing to racial/ethnic disparities. Future research should assess the role of multilevel factors and social determinants of health in contributing to such disparities. An ongoing challenge entails providing risk information to patients transparently to support their informed decisions, because framing of the information may easily become biased. This issue was recently discussed in detail for face VCA transplantation (Smith and Cendales, 2019) and with regard to implicit bias of the informed consent process. Such bias might influence patients’ decisions in a way that makes them too optimistic with regard to their prognosis as well as risks and benefits. Therefore, it is important that doctors are aware of this psychological state of patients that affects their decision as well as of how complex the intertwining of medical ethics and medical practice are (Gilardino et al., 2021, 2022).

### 5.1. Bioethical considerations with special regard to bioethical considerations

Informed consent is best conceived of as a process. Challenges of this process particularly important in the context of VCA transplantation are information itself (which information should be delivered and how understanding could be assessed), the burdens and long-term commitments of VCA regarding immunosuppression and physiotherapy, and the complexity of how to provide new information on outcomes, particularly those referring to risks and complications in order to improve adherence and care management. An expert group (Brocher bioethics working group) recommended consent as a dynamic covenant in order to promote awareness of the importance of transplant recommendations for both patients and caregivers (Magill et al., 2019).

In order to implement such ethical considerations, Chauvet participants discussed a model of initiating the informed consent process by asking the patient what they know and understand particularly about face transplantation in the last meeting. This serves as a baseline for the physician to appreciate the areas in which the patient requires education about the process. Chauvet participants recognized that there is a spectrum of understanding and that there will be different levels of sophistication in different patients. Yet it remains the physician’s responsibility to facilitate informed consent by providing education to meet the level of sophistication of the patient. This process is best achieved by: (1) ensuring that a patient has the capacity to consent (can iterate the risks and benefits and weigh them); (2) conveying the ‘nuts and bolts’ of the procedure; and (3) a longitudinal process with a proposed time of approximately 1 year from the time of initial evaluation, during which the face transplant team should work to understand the patient’s motivations for wanting face transplantation. These recommendations have been discussed at length in the context of face transplantation (Smith and Cendales, 2019; Bound Alberti et al., 2022). But these recommendations present important issues also for the informed consent process in the context of other VCA procedures.

Pediatric VCA is particularly challenging because the effects of the transplant on childhood growth and development are unknown, unlike in the context of kidney, liver, heart, and lung transplantation (Doumit et al., 2014). VCA transplant outcomes may diverge from family and patient expectations and the degree of compliance necessary could become an overwhelming burden (Azoury et al., 2020). Informed consent in the pediatric setting is generally complex (Doumit et al., 2014). Formally, parents or legal representatives have the role of consenting to medical interventions in minors. As there is a general consensus, however, that children should be involved in the consent process according to their decision-making capacity, assessing this capacity at a certain age and in a given situation remains an important area of investigation (Hein et al., 2015). In the VCA context, this assessment can be particularly difficult as treatment options and implications for patient and family are challenging and it might be hard to decide how to best act in the best interest of the child (Azoury et al., 2020). Accordingly, the informed consent procedure needs to be adapted to the pediatric VCA context.

## 6. Conclusion and future challenges

There are fundamental differences between types of VCA, and we have focused much of this manuscript on common psychological dilemmas and future approaches that exist in VCA the majority of patients. Several VCA procedures (i.e., uterus and upper extremity VCA) have emerged as feasible options to provide a functional restoration following traumatic injuries or of infertility. International experience thus far has shown that successful VCA transplantation requires multi-stage multidisciplinary evaluation and follow-up. Candidate selection and evaluator training regarding assessment and ongoing follow-up to address recipients’ post-transplant demoralization, depression, and adherence issues require further refinement to optimize candidate evaluation and follow-up protocols. More broadly, the elaboration of a comprehensive psychosocial framework is needed to provide guidance for individual VCA centers in standardizing their protocols and care procedures. We recommend establishing an international consortium of health care professionals and candidates/recipients (under the auspices of global transplant societies), to facilitate the sharing of experiences and individual perspectives. Use of systematic PROs assessment and follow-up by the recommended interdisciplinary transplant consortium may foster the identification of risk factors and patients’ needs outside of routine clinical care, where patients’ needs are often not addressed. Ultimately, we envision that such a consortium will greatly improve the exchange and networking among VCA providers and patients through international research that will support the advancement of psychosocial evaluation of VCA.

## Author contributions

MK participated in research design, the writing of the paper, performance of the research, and contributed new insights. SJ-G, EG, and GW-F participated in the writing of the paper, performance of the research, and contributed new insights. All authors contributed to the article and approved the submitted version.

## Conflict of interest

The authors declare that the research was conducted in the absence of any commercial or financial relationships that could be construed as a potential conflict of interest.

## Publisher’s note

All claims expressed in this article are solely those of the authors and do not necessarily represent those of their affiliated organizations, or those of the publisher, the editors and the reviewers. Any product that may be evaluated in this article, or claim that may be made by its manufacturer, is not guaranteed or endorsed by the publisher.

## Abbreviations

AST: American Society of Transplantation
FTx: face transplantation
HRQOL: health-related quality of life
OPTN: Organ Procurement and Transplantation Network
PROs: patient-reported outcomes
QOL: quality of life
UETx: upper extremity transplantation
UTx: uterine transplantation
VCA: vascularized composite allotransplantation
